# Indigenous Perspectives on the Co-management of a Regional Conservation Area in the Peruvian Amazon

**DOI:** 10.1007/s00267-025-02310-2

**Published:** 2026-02-20

**Authors:** Natalia Arcos Cano, Michael P. Gilmore, Elizabeth Benson, Diego Valderrama, David Dimitrie, Brian M. Griffiths

**Affiliations:** 1 Department of Environmental Science and Policy, George Mason University, 4400 University Drive, Fairfax, VA USA; 2 School of Integrative Studies, George Mason University, 4400 University Drive, Fairfax, VA USA; 3 OnePlanet, Inc., 6712 Wooden Spoke Rd, Burke, VA USA; 4 Jirón Napo, Iquitos, Peru; 5 Detroit Zoological Society, Royal Oak, MI USA; 6 The Earth Commons—Georgetown University’s Institute for Environment & Sustainability, 3700 O St. NW, Washington, DC USA

**Keywords:** Conservation, Culture, Governance, Loreto, Maijuna, Protected area

## Abstract

Growing awareness of the critical role Indigenous Peoples and Local Communities (IPLCs) play in biodiversity conservation has underscored the need to shift conservation practices towards empowering IPLCs, supporting their land rights, traditional practices, and facilitating their political involvement. Despite IPLCs governing over 32% of global land, historically these communities have faced systemic marginalization and violence in the name of conservation. In response, international calls to action and policies have aimed to enhance IPLC participation in environmental governance through mechanisms like co-management. Adaptive Co-management (ACM) emerges as a promising approach, combining adaptive management’s flexibility with co-management’s collaborative principles. This study evaluates the ACM framework within the Maijuna-Kichwa Regional Conservation Area (MKRCA) in the Peruvian Amazon, established to protect the ancestral lands and biocultural resources of the Maijuna and Kichwa communities. Using a Community-Based Participatory Research (CBPR)-informed approach, we conducted interviews with 36 community members to assess their perspectives on the MKRCA’s co-management. Findings reveal significant improvements in resource abundance and community safety but also highlight issues with governmental support, communication, and equitable participation. Highly engaged participants voiced the strongest criticisms, underscoring how participation level shaped perceptions of governance. Recommendations were derived from participant perspectives but synthesized by the authors rather than fully co-developed. The study emphasizes the need for continuous evaluation and enhanced stakeholder engagement to optimize ACM’s effectiveness, offering culturally responsive recommendations to strengthen the MKRCA’s management and achieve its conservation objectives.

## Introduction

Over 32% of global land is either owned or governed by IPLCs in some way (Fa et al., 2020), the majority of which is composed of land in good ecological state, comprising a large proportion of the International Union for Conservation of Nature’s (IUCN) Key Biodiversity Areas (WWF et al. 2021). Historically, IPLCs have been marginalized and subjected to systemic discrimination and abuse, from enslavement and displacement to forced assimilation, with these abuses often happening in the name of biological conservation (Esparza 2006; IWGIA 2020; Luoma 2023; Smallwood et al. 2021). The recognition of the violence inflicted upon IPLCs for over 150 years in the name of conservation has led to the establishment of different international calls for action aimed at incentivizing the effective participation of IPLCs in the governance and protection of their territories and resources (International Labour Organization (ILO) 1989; IUCN and Conservation International 2022; Jaireth and Smyth 2003; United Nations 2007).

IPLC participation can be achieved through the implementation of participatory governance mechanisms, such as the co-management of protected areas and natural resources (Armitage et al. 2011; Cadman et al. 2022; DePourcq et al. 2015). However, co-management can have many issues, as oftentimes institutions are not prepared to provide the level of support and empowerment necessary to achieve the required community engagement for effective co-management, and preexisting social hierarchies can hinder the establishment of an equal partnership (Armitage et al. 2008; Castro and Nielsen 2001; DePourcq et al. 2015; Tuan et al. 2017).

One approach proposed to address the governance of complex social-ecological systems (SES) is adaptive co-management (ACM). This participatory governance mechanism provides a sense of flexibility that is hard to achieve under other Western resource management systems (Armitage et al. 2008; Plummer et al. 2012). ACM merges the principles of adaptive management, with its emphasis on learning-by-doing and enhancing the system’s adaptive capacity, and those of co-management, which seeks to bridge the gap between governments, stakeholders, and diverse knowledge systems to address common challenges (Armitage et al. 2008; Plummer et al. 2012). This approach ensures that solutions are tailored to the unique contexts and needs of the many stakeholders involved. Continuous evaluation and feedback opportunities within the governance structure are an essential aspect of successful ACM, as they allow managers to continuously assess and adapt strategies in response to changing needs and conditions of the SES (Armitage et al. 2008; Cox et al. 2020; Plummer et al. 2012).

In the last two decades, the Peruvian government has taken a decentralized approach to its land governance, aiming to better respond to regional needs through the creation of direct participation channels between the government and local communities (Agrawal and Ribot 1999; Libert-Amico and Larson 2020). Regional jurisdictions are granted exclusive authority over territorial demarcation, sustainable use and conservation of forest resources and biodiversity, natural resource management, and administration of reserves and Regional Conservation Areas (RCAs) (Agrawal and Ribot 1999; Ministerio de Economía y Finanzas 2019). RCAs are classified as areas of direct use, meaning resource extraction and other activities are permitted as long as they fall within the objectives and stipulations of the area’s management plan. The governance system of these RCAs can vary. Some, such as the Maijuna-Kichwa Regional Conservation Area (MKRCA), are under co-management schemes. However, the effectiveness of the management plan and governance in meeting the RCA’s objectives is rarely evaluated.

Recognizing the importance of project evaluation within an Indigenous context (Bowman et al. 2015; Luoma 2023; Tauli-Corpuz 2016) and the value of community perspectives within adaptive co-management schemes (Armitage et al. 2008; Ruitenbeek and Cartier 2001), makes evaluation more critical. In this study, we conducted semi-structured interviews with Maijuna community members to understand their perspectives on the management of the MKRCA, and test the hypothesis that the Maijuna understand and support the co-management structure and process that is currently in place. The results of this research provide relevant stakeholders with insights to optimize and enhance future management efforts to achieve the biocultural conservation objectives laid out since the MKRCA’s establishment. This collaborative management structure forms the foundation of community-based conservation and sustainable development systems that are models for the rest of the Amazon rainforest, if they can be managed correctly.

## Methods

### Study Site

**Fig. 1:**
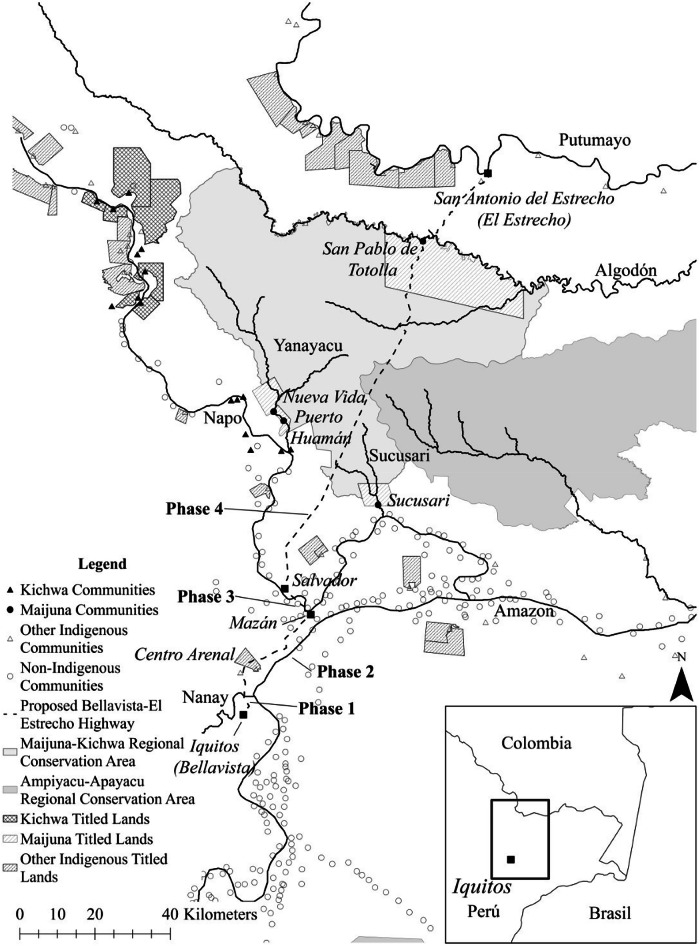
Map showing the Maijuna and Kichwa communities, the Maijuna-Kichwa Regional Conservation Area (MKRCA), and the proposed Bellavista-Mazán-Salvador-El Estrecho Highway, connecting the city of Iquitos with the town of San Antonio del Estrecho (El Estrecho) on the Putumayo River

Established in 2015 in the department of Loreto, the MKRCA is an RCA spanning 391,039.82 hectares of tropical rainforest between the Napo and Putumayo rivers (Fig. 1). As part of a co-management structure, the MKRCA is governed through a Management Committee (Comité de Gestión) that brings together representatives from the four Maijuna communities through their federation, FECONAMAI, and from the over 23 Kichwa communities part of the MKRCA buffer zone through their federation, FECONAMNCUA, local guard organizations (OLVs), NGOs, and the Loreto Regional Government (GOREL). The Committee operates through a General Assembly and a smaller Directing Council (president, vice president, technical secretariat, and members), with leadership elected every two years. This structure provides the principal forum for decision-making, accountability, and implementation of the MKRCA Master Plan (Autoridad Regional Ambiental Loreto 2020). Although not all community members participate directly in Management Committee meetings, they are engaged in co-management through their voluntary work in the OLVs, patrolling the MKRCA and its buffer zone as a way to monitor and identify illegal mining, fishing, poaching, or logging activities that can impact the ecological integrity of the MKRCA and the wellbeing of its beneficiaries.

Many aspects contribute to the ecological significance of the MKRCA, from containing the headwaters of seven tributaries to two of the largest rivers in the Peruvian Amazon and containing a unique and previously undiscovered high-terrace forest ecosystem, to being the home of a significant number of threatened and endangered plant and animal species, some of which are new-to-science or yet to be described (Gilmore et al. 2010). Additionally, the MKRCA contains an unprecedented density of mineral licks, which provide essential nutrients and social benefits to various mammal and bird species, including the Brazilian tapir (*Tapirus terrestris*), the white-lipped peccary (*Tayassu pecari)*, the collared peccary *(Pecari tajacu*) and the lowland paca (*Cuniculus paca*) (Griffiths et al. 2024, 2020). The conservation of these species and ecosystems is the first objective of the MKRCA Management Plan, followed by the sustainable use of natural resources for economic benefits and to revitalize the culture of the Maijuna and Kichwa (Autoridad Regional Ambiental Loreto 2020).

The Maijuna, one of the 32 Indigenous groups in the department of Loreto, are a western Tucanoan people that have occupied an area between the Napo and Putumayo rivers for millennia (Bellier 1993). Currently divided into four different communities (Puerto Huamán, Sucusari, Nueva Vida, and San Pablo de Totolla) with a total population under 600 people, the Maijuna are among the smallest and most vulnerable Indigenous groups of Peru (Gilmore et al. 2010). The Maijuna hunt, fish, farm, and extract a variety of forest resources from their ancestral lands for both subsistence and income generation (Gilmore et al. 2010). Although there are four Maijuna communities within the MKRCA, fieldwork was conducted in three of them (Puerto Huamán, Sucusari, and Nueva Vida). San Pablo de Totolla, the most geographically isolated community, could not be included due to mobility, accessibility, and time constraints.

### Data Collection

In this study, we employed a Community-Based Participatory Research (CBPR)-informed approach, a subset of action research methodology commonly used for identifying and addressing local issues through collaboration and trust-building between researchers and members of affected communities (Christopher et al. 2008; Duke 2020; Holkup et al. 2004). While not all stages of CBPR could be fully implemented due to structural and contextual constraints, this study was guided by the CBPR principles of reciprocity, relevance, and respect. The CBPR-informed approach was applied to a case study design, which allows researchers to evaluate a group of people after an intervention has taken place to assess the impact of said intervention (Bernard 2011). In this case, the interventions being evaluated were the creation and ongoing co-management of the MKRCA from 2015 to the time of the evaluation.

With the help of Maijuna community leaders, we identified and selected a set of 36 participants through maximum variation purposeful sampling (Bernard 2011; Rasmus 2014), which allows for high participant heterogeneity and the identification of patterns among a wide range of cases (Rasmus 2014) thus providing a more holistic understanding of Maijuna perspectives. Selected participants were adult Maijuna community members of varying ages, genders, and levels of community engagement.

The sampling strategy aimed to maintain a balanced gender representation (male and female), targeting six interviews per gender in each of three Maijuna communities, for a total of 36 semi-structured interviews. Within each gender group (M or F), three categories of individuals based on community engagement were targeted: G1 represented individuals with the highest level of engagement, G2 included those moderately involved in community affairs, and G3 encompassed individuals who had minimal engagement with community matters (Table 1).

**Table 1:** The number of participants in each category, as well as the age range and median age for each group

Category	*N*	Age range	Median age
MG1: Men, High Engagement	8	33–70	45
MG2: Men, Moderate Engagement	6	35–70	50
MG3: Men, Low Engagement	6	23–71	56
FG1: Women, High Engagement	5	30–54	39
FG2: Women, Moderate Engagement	5	45–67	51
FG3: Women, Low Engagement	6	25–72	31

During the data collection process, challenges emerged, particularly with gender and age diversity. We faced difficulties in recruiting women with high levels of community engagement due to the patriarchal nature of the Maijuna culture, resulting in a limited number of highly involved women willing to participate in interviews. The age diversity within the study was also affected. Younger community members (18-30) were less inclined to participate in interviews, while individuals highly involved in community affairs tended to be predominantly from older generations. One interview with a Kichwa individual from the community of Nueva Floresta was included in this study; he was visiting one of the Maijuna communities during the fieldwork period and, due to his level of community engagement, was categorized as G1.

In efforts to alleviate these issues, recognizing the importance of creating a comfortable environment for women to speak freely, we scheduled interviews during times when husbands were not at home, allowing for a more relaxed setting and increased willingness among women to participate. We also actively engaged in the activities that women were undertaking at the time of the interviews. This included participating in daily tasks or communal activities fostering a sense of familiarity and trust. However, due to the nature of the communities, where a significant proportion of younger individuals were engaged in hunting, subsistence activities, or pursuing further studies outside the community, it remained challenging to match the number of older subjects with younger ones.

Fieldwork, conducted between June and July of 2023, began with an introductory meeting in each community to familiarize the community with the researcher, provide appropriate information about the study, and seek the community’s prior informed consent (PIC) (Bernard 2011). After obtaining community approval, pilot interviews (Abdul Majid et al. 2017; Sampson, 2004) were performed with select community members, and the interview questionnaire was adapted based on their feedback and recommendations. Then, PIC was sought from the individuals identified in each community before conducting approximately 1-hour individual semi-structured interviews with them (Bernard 2011; DiCicco-Bloom and Crabtree 2006) (see Supplementary Information).

Per the flexibility required when conducting CBPR (Collins et al. 2018; Harrison et al. 2001; Holkup et al. 2004), different measures were adopted along the way to ensure the participants’ comfort throughout the research process. These measures included the availability of a Maijɨki translator for individuals who found greater comfort in communicating in their native language instead of Spanish. Recognizing the significance of cultural and linguistic nuances, the translator’s presence aimed to facilitate open and meaningful communication.

### Community Participation and Partnership

This research was conducted in partnership with the Federación de Comunidades Nativas Maijuna (FECONAMAI), the formal representative body of the four Maijuna communities. The research focus stemmed from concerns expressed by Maijuna leaders about limited government engagement in MKRCA management and the absence of evaluation since its establishment. The semi-structured questionnaire was reviewed and revised with input from community members and OnePlanet Peru staff, who have long-term relationships with the Maijuna. Research authorization was sought from FECONAMAI leaders, who convened village assemblies where the project was explained, consent procedures discussed, and dissemination preferences determined. Community members requested oral presentations as the preferred format for returning results, and these were later delivered in each community and to the MKRCA Management Committee. Although literacy and connectivity barriers limited participation in formal data analysis and writing, emerging themes were discussed informally during fieldwork to check interpretations.

### Ethics and Data Handling

In alignment with IRB approval (IRBNet number: 2053918-1), prior informed consent was obtained verbally due to literacy constraints. At the start of each interview, participants were asked for permission to audio-record and reminded that they could decline or rescind their consent at any time; one participant withdrew consent and their data were excluded. Recordings were transcribed using Cockatoo software, which encrypts files, does not share data with third parties, and allows users to delete files at any time. While explicit consent for AI-based transcription was not obtained—representing a limitation in terms of Indigenous data sovereignty—care was taken to minimize risks by relying on encrypted, non-commercial storage. Because of literacy and connectivity barriers, returning transcripts individually was not culturally or practically feasible; instead, results were validated and returned collectively through community assemblies and oral presentations, in the format requested by participants.

### Data Analysis

The data collected was first transcribed using the AI-powered transcription service Cockatoo (Cockatoo Inc. 2024). Following this initial transcription, and in alignment with Braun and Clarke’s (2006) six-phase approach to thematic analysis, each transcript was carefully reviewed. During this review, notes were taken while listening to the respective interviews to correct any mistakes and further familiarize the researcher with the data. The thematic analysis process began with multiple scans of each transcript, where the data was coded into different categories based on persistent concepts. Deductive thematic analysis guided the project, with the objectives and general management framework laid out in the MKRCA’s management plan. However, as Braun and Clarke (2012) noted, the coding process often incorporates inductive and deductive methods. In this project, the development of subcodes was primarily derived from the data content itself.

After coding all the documents in the qualitative data analysis software Delve (Twenty to Nine LLC 2024), themes that were coherent and relevant to the research question were identified per Braun and Clarke (2006). This process involved organizing the codes into potential themes and ensuring they told a comprehensive story related to the research question. Once the initial themes were identified, they were reviewed along with the associated codes to ensure that the themes accurately reflected the data and that no significant information was overlooked. Finally, we selected quotes that clearly illustrate the identified themes and ground them in community members’ voices.

## Results

The thematic analysis of the semi-structured interview data revealed five primary themes crucial to understanding community members’ perceptions of the MKRCA’s co-management effectiveness and its alignment with their culture and traditional practices. These themes are labeled as “Co-management,” “Cultural Conservation,” “Regional Conservation Area,” “OLVs,” and “Dreaming and Design” (Table 2).

**Table 2:** Themes resulting from the thematic analysis of the semi-structured interview data, along with their associated codes and subcodes

Themes	Primary codes	Secondary codes
Co-management	Trust and Reliability	
	Community compliance	
	Communication	Management Committee
	Regional Government	Visits
		Implementation and provisions
	NGOs	
	Maijuna-Kichwa Relations	
Regional Conservation Area	Understanding	“I don’t know”
	Opinion	Resource Use
		Dissatisfaction
		Access to Initiatives
	Changes	Safety
		Abundance
		Organization
OLV	Understanding	
	Opinion	
	Enforcement	
Cultural Conservation	Intergenerational Knowledge Transfer	Youth
	Empowerment	Ownership
		Unity
		Pride
	Máíjìkì Preservation	
	Cultural Conservation	
Dreaming and Design	Recommendations	
	Support needed	Education
		Health
		Financial
	“We don’t want the highway”	

The table provides a clear overview of how the data was categorized, offering insight into the persistent concepts and underlying patterns identified during the analysis.

### Co-management

Co-management in the RCA’s management plan is characterized by the active participation of local actors in the MKRCA’s management (Autoridad Regional Ambiental Loreto 2020). When asked who they perceive as the MKRCA manager, participants’ answers varied from “Who manages? Everyone! All of us!” to the “RCA is the State.” Some even associated FECONAMAI, the Maijuna Federation, with the head of the MKRCA. Notably, even participants with high levels of community engagement had trouble identifying the manager of the Area.

Eleven out of 36 (30.5%) community members expressed a lack of trust in the regional government in regards to co-management. This distrust was exacerbated by the government’s failure to follow through on promises: “For example, they say that there is a project that offers certain benefits, but in the end, these projects do not work because they are not fulfilled.” The same number of participants mentioned issues with community compliance, where some members did not adhere to RCA rules: “Now things are changing a little, that’s why I’m saying that some people don’t listen, and that still bothers me. They don’t follow the rules. For example, I’m a guard; I watch over things, and others take game meat, sell it, leave, and take more again. That’s not how it should be; we need to respect the rules and follow what is said.”

Communication, a critical aspect of co-management, emerged as a significant issue as well. Twelve community members felt uninformed about ongoing activities and expressed a desire for more transparency and involvement. This lack of information contributed to reduced motivation to comply with regulations and patrolling efforts, as some individuals did not see the broader impact of their efforts: “That’s why I also think it’s pointless, I say it’s pointless to comply with them because they don’t comply with us by doing what they said they would do.” Participants suggested more community involvement in management meetings that take place twice per year, noting that most attendees are invited merely as witnesses, without the ability to contribute to the agenda: “And how are we going to participate if we only came to watch? We have something to propose as well, to contribute something, even if it’s not much.”

Regarding their co-management partners, 14 community members expressed discontent with the regional government and greater affinity with the NGOs that partner with communities in the region: “I think they should come themselves to give us workshops, to give us value, motivation, to guide us together, to supervise how to do things, like them as leaders, chiefs, as the regional government so that they can also see what the issues are in the MKRCA and how we can improve them. They should see and understand, not just tell us to go to some NGO that will send help…”. Another sentiment was the community members’ dissatisfaction with the lack of government presence in the MKRCA, highlighting the brevity of their visits.

The relationship with the Kichwa, a code present in 16 transcripts, varied, as some question why additional Kichwa communities continue to join the management committee without apparent benefit, highlighting issues with communication. Conversely, others admire the Kichwa for their strong ties to cultural traditions, viewing them as exemplars for Maijuna language conservation. The use of “siblings” by some participants in reference to the Kichwa and Maijuna people indicates affinity and alliance.

### Regional Conservation Area

Understanding of the MKRCA varied among community members. While some, particularly women (80% of the women interviewed), expressed uncertainty about different aspects of what the MKRCA entailed, others could explain its origins and purpose. Generally, the MKRCA was seen as a means to protect their land for future generations, with iterations of the word *cuidar*, which translates to “take care of” or “protect,” being used in 58.3% of interviews.

Opinions on the MKRCA’s impact were mixed. 22 out of the 36 participants (61.1%) were grateful for the resource management strategies that had led to an abundance of resources and access to initiatives like beekeeping and handicraft sales: “I feel happy about the RCA. I feel good because we also benefit from it and are doing well.” However, eight other participants (22.2%) saw unequal access to these initiatives as a point of contention, with some members being excluded due to a lack of understanding or interest: “Sometimes, benefits arrive for the community. But the benefits only reach a certain group.” Significant positive changes attributed to the MKRCA included reduced illegal resource extraction (97.2% of interviews), improved quality of life (83.3%), better community organization (33.3%), and increased safety (13.8%): “Why is there so much wood? That’s why. Why are there so many fish? That’s why, because we are taking care of it. That’s why we are here; we are the owners of this.” However, resource use practices remained contentious, with the underlying feeling that their efforts in monitoring the RCA did not yield sufficient benefits. Some community members advocated for a complete ban on extraction: “…by guarding here, or watching over this RCA, I don’t even go to the forest to hunt for myself, for my benefit, I’m taking care of it for others. I’m not going to take care of it so others can come and eat, and that’s why I decided we shouldn’t let anyone enter…”. Others wished for more access to resources without facing penalties: “Well, as I say, right now some of us want to log to support the needs we have…”

### OLVs

The theme of OLV emerged as a central component of community-led efforts to monitor and protect resources within the MKRCA. The OLV plays a crucial role in enforcing regulations, conducting patrols, and ensuring compliance with conservation guidelines. Most community members (69.4%) expressed their active participation in OLV activities, such as monitoring, reporting violations, and implementing sustainable resource management practices. The OLV is depicted as a key mechanism for community empowerment, self-governance, and environmental stewardship: “Particularly, the community of Nueva Vida is very interested and is actively practicing through its control and vigilance, which is the most important thing and is part of the regional government. They are volunteers working on their own initiative, becoming more and more aware and getting more and more involved, and eventually, almost the entire community is involved.”

However, as mentioned previously, some community members lack the motivation to participate in the OLV efforts due to the lack of direct benefits and interference with their subsistence practices. One member expressed this sentiment clearly: “…if I have to do my harvesting today and it’s my turn to do my patrol, who pays me? What do I prefer? To do my harvesting because it benefits my family and my children, and this doesn’t. This only benefits the GRAM [*Gerencia Regional Ambiental or Regional Environmental Management*], right?” Additionally, there is a strong emphasis (55.5%) on the need for provisions, food, and transportation: “We need a little more support for the patrol efforts, such as equipment, for example… a boat, to improve mobility for patrols, right? … This should be strictly for the patrols. It should be at their disposal, with fuel available, and people will be able to do their patrols without any issues.” The collaboration between the OLV, community members, government agencies, and external organizations to manage and protect the MKRCA effectively was also highlighted regarding the enforcement of resource management policies: “If we ever need assistance, we can call; we have the numbers of the relevant authorities. We call them; they can intervene because we don’t intervene ourselves.”

### Cultural Conservation

The theme of cultural conservation emerged prominently across the transcripts of various participants (63.8%). When asked what is important to conserve within their communities, participants expressed admiration for their traditional customs: “Yes, look, the customs, the customs. Do you know why I talk about customs? The customs we have as Maijuna are very beautiful… “ Some emphasized the need to pass down cultural knowledge to younger generations to prevent its loss: “If we remind the children, you know, if you show interest in the children, it is easy for them to learn. And then we teach them so they can continue the next generation because if they don’t dedicate themselves, it dies out, you know?”

Participants also recognized the importance of empowerment in maintaining and incentivizing cultural conservation: “Maybe we can find an incentive, some economic means for the elders who speak the language before they pass away, so they can also give their time and teach.” They stressed the importance of valuing the elders’ time and effort. The Kichwa were used as inspiration due to their seemingly stronger connection with their cultural roots: “I have observed in the upper Napo, the Kichwas. Little children speak in their own language… From a young age in school, singing their national anthem in their own language. How beautiful! I would also like the Maijuna people to be the same, yes.”

### Dreaming and Design

The “dreaming and design” theme showcases community members’ aspirations for a better future and their strategic planning to achieve their goals. This concept is reflected in the community’s vision for improvement, such as seeking support for sustainable projects, advocating for better infrastructure, and planning for cultural preservation. While not explicitly mentioned as “dreaming and design,” the concept is evident in 33 out of the 36 transcripts (91.6%). Community members highlighted their desire for more projects that benefit everyone, emphasizing the importance of education: “I hope there will be more projects that benefit the community for everyone so that we can educate our children so that our children can move forward and become something in life. They need to study to prepare themselves. That’s what I want for the future, for young people to study and for my children to study as well.” Additionally, participants expressed the desire for improved living conditions that come with economic development and better infrastructure as incentives for their OLV efforts: “You take care of things, but you earn a monthly salary, you get your money, you can send your children to school, you can fix your houses… An improved home with your latrines, complete service, sidewalks so you don’t step in mud, and a main port. All that is an improvement so that the community feels happy.”

Health was also a critical concern, with aspirations for better healthcare access and support from NGOs: “First of all, let’s talk about health because it’s the most important thing. If you are not healthy, you can’t expect anything. In ten years, I want things to be different. When everyone is sick, no one can work. Perhaps health, medication, maybe from other NGOs that want to provide support, they can provide support through first aid.” The design aspect is implied in discussions about organizing workshops, implementing projects, and seeking external assistance to enhance livelihoods and conservation efforts. There is a strong desire for economic stability to reduce dependence on forest resources: “Support us here so that the economy provides for us. We wouldn’t go to the forest all the time. No, we wouldn’t always be looking for things to sell. Why would we go to the forest if we have access to the economy? Only to eat, nothing more.”

The construction of the Bellavista-Mazán-Salvador-El Estrecho highway presented a significant obstacle to these dreams for some respondents. This megadevelopment project is scheduled to connect Iquitos, the main city of the Loreto region, with the Colombia border along the Putumayo River (Vilela et al. 2020). This highway would bisect the MKRCA, along with the titled lands of the Maijuna, and the headwaters of the Sucusari and the Algodoncillo Rivers, primary water sources for the Maijuna people (Gilmore et al. 2010). Though the megadevelopment project was neither introduced by the interviewer, nor included in the interview questionnaire, twelve out of the 36 (33.3%) community members raised the subject, expressing strong opposition due to concerns about its potential negative impacts on the environment, wildlife, and their way of life. They viewed this infrastructure project as a significant threat that could lead to deforestation, loss of biodiversity, and increased access for outsiders and colonists, potentially resulting in conflicts. Community members feared that the highway would derail their aspirations for a better future, emphasizing their commitment to protecting their lands and resources from such external threats: “Above all, regarding the RCA, we are going to take care of it, we will take care of it until the end, until we achieve it. We don’t want the highway”.

## Discussion

Our analysis results provided crucial insights into community members’ perceptions of the MKRCA’s co-management effectiveness and its alignment with their culture and traditional practices. Comparing these results with the conditions for successful ACM (Armitage et al. 2008) (Table 3) shows that there are both strengths and challenges in the governance of the MKRCA’s co-management framework.

**Table 3:** The conditions for ACM success as defined by Armitage et al. (2008), an evaluation of whether these conditions are currently being met by the MKRCA’s ACM, and corresponding recommendations for each condition

Conditions for ACM success	Status	Recommendations
Well-defined resource system	Met	
Small-scale Resource Use Context	Unmet	- Increased risk-sharing among stakeholders - Stronger support from GOREL in the fight against highway development
Clear and Identifiable Entities with Shared Interests	Unmet	- Increase length and number of visits by regional government officials to the communities to enhance narrative congruence
Reasonably Clear Property Rights to Resources of Concern	Unmet	- Enhanced government action to honor their commitments promptly and effectively as to increase trust
Access to Adaptable Portfolio of Management Measures	Partially met	- Increase community influence in the decision-making process - For educational programs to be delivered in culturally resonant ways and tailored to the participants’ learning needs
Commitment to Support a Long-term Institution-Building Process	Partially met	- Increase government interaction and support to the communities - Focus on development of self-sufficient management efforts
Provision of Training, Capacity Building, and Resources for Stakeholders	Unmet	- Increase financial support from government - Enhance adaptability and evaluation of management - Expand and enhance learning and capacity building opportunities
Key Leaders or Individuals Prepared to Champion the Process	Partially met	- Implement youth and women empowerment initiatives - Promote inclusive local leadership - Cultural sensitivity training for government officials
Openness of Participants to Share and Draw Upon a Plurality of Knowledge	Partially met	- Expand and improve educational programs - Enhance stakeholder collaboration - Incorporate non-expert knowledge into the decision-making process - Promote information dissemination
National and Regional Policy Environment Explicitly Supportive of Collaborative Management Efforts	Partially met	- Address conflicting interests regarding the highway development project - Empower and support local communities in their fight against the highway - Establish clear objectives and explicit policy support for collaborative processes and multi-stakeholder engagement

The first of Armitage et al.‘s (2008) conditions is a well-defined resource system. The MKRCA fully meets this specific condition, delineating 391,039.82 hectares. There are also pre-established resource use guidelines set forth by the management plan to avoid overhunting and commercial resource extraction (Autoridad Regional Ambiental Loreto 2020). The second condition for success is a small-scale resource context. The MKRCA is finite and can be considered a small-scale system.

Regarding conflicts of interest, it is essential to acknowledge that Peru’s central government has an interest in the development of the Bellavista-Mazán-Salvador-El Estrecho highway (Fig. 1). The construction of this highway represents one of the primary conflicts confronting the MKRCA system. This proposed megadevelopment project not only jeopardizes the ecological integrity of the MKRCA, but it would undermine the livelihoods of communities that depend on the MKRCA for sustenance and cultural preservation (Gilmore et al. 2010), all while eroding the trust relationships among stakeholders (Koch et al. 2023). Within a group such as a management committee, the collective co-creation of understanding of a problem and its solutions is known as narrative congruence (Koch et al. 2023). Trust is a fundamental component of narrative congruence in ACM, and once lost, it is challenging to restore (Armitage et al. 2008; Cox et al. 2020; Koch et al. 2023).

The third condition, having clear and identifiable entities with shared interests, underscores the significance of narrative congruence, belonging, and local participation within ACM systems. As described by Armitage et al. (2008), the general lack of connection to “place” among certain stakeholders, in this case regional government officials, or other non-indigenous actors involved with the MKRCA, can lead to a deficit of trust among stakeholders. This arises from the fact that the MKRCA holds ancestral significance for the Maijuna and Kichwa communities. Due to the perceived disconnect between the regional government entities and the communities, community members want a more prolonged and direct presence and support from the regional government. Research indicates that “the frequency of interaction among diverse stakeholders influences the building of a mutual understanding of a problem” (Koch et al. 2023, p. 2), suggesting that longer, more frequent interactions between GOREL and the communities could foster trust and enhance narrative congruence regarding the MKRCA’s current challenges.

Condition four, having reasonably clear property rights to resources of concern, underscores the imperative of clearly and comprehensively communicating stakeholders’ rights and responsibilities. Plummer et al. (2012) promote positive collaboration in ACM, illustrating how landowners and resource stewards are more inclined to sustain biodiversity if their contributions to conservation efforts are acknowledged. In the context of the MKRCA, members of the local guard organizations express a sense of guardianship over the land “for someone else,” as they perceive a lack of direct recognition for their commitment to safeguarding the MKRCA’s resources. Interviewees frequently lamented the perceived absence of support from GOREL in fulfilling the promise of food, fuel, and other supplies essential for sustained patrolling efforts. This failure to provide the promised support contributes to a feeling that the government is not meeting its responsibilities, further eroding trust and complicating the co-management process.

Condition five, access to adaptable portfolio of management measures, posits that participants in ACM processes should be able to explore and implement diverse management measures or tools to achieve the agreed-upon objectives or address any challenges or needs within the ACM system. From education to technologies and regulatory tools, stakeholders and participants in ACM should be allowed to participate in the knowledge development process and contribute to its implementation (Armitage et al. 2008; Cox et al. 2020; Plummer et al. 2012). However, in the case of the MKRCA, this ideal condition encounters practical limitations. While avenues for participation exist, such as involvement in management committee meetings and participation in the OLV volunteer programs (Autoridad Regional Ambiental Loreto 2020), community members lack direct influence over agenda-setting or rulemaking within the MKRCA’s governance process. This diminishes their ability to contribute substantively to decision-making. While participants discussed workshops for learning about governance, many respondents – particularly women and young adults – were not able to describe how the governance structure works. These discrepancies underscore the importance of constant evaluation of the achievement of the goals of the MKRCA (Armitage et al. 2008; Cox et al. 2020; Plummer et al. 2012). Notably, highly engaged individuals (G1) most often voiced the strongest criticisms of MKRCA governance, whereas less engaged participants (G2 and G3) articulated fewer detailed concerns, suggesting that participation level shaped perceptions of co-management.

The sixth condition for ACM success is a commitment to support a long-term institution-building process. The presence of this sixth condition in the MKRCA’s ACM can be witnessed when hearing the community members speak about what they want their children and grandchildren to grow up around or express the deep sense of ownership and relation they feel toward their ancestral lands. While the community’s dedication is undeniable, the government’s commitment is questionable, as reflected in their lack of interactions with the communities and the MKRCA as a whole. Additionally, NGOs operate on a project basis, and although some have shown long-term commitment, it would be beneficial for the capacity-building and management efforts to be less dependent on NGOs since their funding is not always consistent.

The seventh condition for successful ACM, the provision of training, capacity building, and resources for stakeholders, is not fully present in the MKRCA. As previously stated, community members often have to borrow from their own resources or ask for NGO assistance to participate in ACM processes, such as attending meetings, scheduling meetings with government officials, or patrolling with the OLVs. Due to the lackluster financial support the central government provides, regional governments are often underfunded and can’t provide the required assistance to RCA co-management partners (Rodriguez-Ward et al. 2018).

The eighth condition is the presence of key leaders or individuals prepared to champion the process. Community leaders coming together to champion their community’s interests was the original driver for the establishment of the MKRCA (Gilmore et al. 2010). However, elders predominantly assume these leadership roles, with limited interest from younger community members to participate or eventually take over these prominent positions as elders step back. Implementing youth empowerment initiatives is critically important for maintaining the MKRCA’s ACM system. In addition to community leadership, it is crucial to consider the administrators on the government front. Ensuring that those in leadership positions within the regional government exhibit a commitment to and understanding of the MKRCA’s historical, cultural, and ecological significance to the Maijuna and Kichwa is essential. These leaders must champion the management process with this perspective in mind.

Condition nine is an openness of participants to share and draw upon a plurality of knowledge. The MKRCA’s co-management structure aims to facilitate collaboration between various stakeholders, including the Maijuna, the Kichwa, governmental bodies, and NGOs. Despite this acknowledgment, community members expressed concerns about their limited influence over decision-making processes, particularly regarding agenda-setting and rulemaking within the governance structure. To successfully meet this condition, the government needs to improve its communication with the communities and other stakeholders. This could include longer and more frequent visits, asking for input and discussion on agenda items, and distributing an easily understandable log of the actions taken at each management committee meeting so attendees can disseminate the information, among many other things. Ensuring that people are better informed is crucial for effective participation and engagement. Similar challenges have been documented elsewhere, such as Indigenous co-management in South Australia where participation is often limited to plan-level decisions rather than true co-governance (Gienger & Nursey-Bray 2025), and in Chile where ecosystem-based management commitments remain fragmented in practice (Gelcich et al. 2018).

The final condition for ACM success is that the national and regional policy environment is explicitly supportive of collaborative management efforts. The establishment of the MKRCA itself reflects a level of support from the Peruvian government for multi-stakeholder engagement in conservation initiatives. The decentralized approach to governance, as evidenced by the creation of RCAs and the involvement of Indigenous communities in management committees, suggests a willingness to distribute functions across organizational levels. However, issues such as insufficient financial support from the central government and conflicting development projects, like the proposed Bellavista-Mazán-Salvador-El Estrecho highway, may undermine the effectiveness of collaborative management efforts. To enhance the likelihood of success, clearer objectives, the provision of resources, and consistent support from policy sectors at both national and regional levels are needed. This includes ensuring that development projects go through the appropriate, legal consultation and prior-informed consent processes before being implemented.

It is also important to note that while recommendations were grounded in participant perspectives, their final formulation was synthesized by the authors rather than co-developed in full partnership with community members.

### Limitations

This study has two primary limitations. First, fieldwork was conducted in three of the four Maijuna communities; San Pablo de Totolla, the most geographically isolated, could not be directly sampled due to accessibility and time constraints. As a result, perspectives from this community may be underrepresented, particularly given its relative isolation and the proposed highway that would pass through its territory. Second, while this research followed a CBPR-informed approach, structural barriers such as limited literacy, connectivity, and competing community priorities prevented direct participation in data coding and manuscript preparation. These constraints limited involvement across all stages of the research, though the study sought to uphold CBPR principles by involving community members in design, implementation, and dissemination.

## Conclusion

Based on our analysis and these critical conditions for ACM success, we propose a series of action items for improvement of governance of the MKRCA, derived from participant perspectives and concerns but synthesized by the authors rather than formally co-developed or validated line-by-line with participants. These recommendations draw inspiration from the five-step resilience-based framework developed by Galappaththi et al., (2022).

1. *Enhanced Engagement through More Frequent and Extended Visits:* Increase the frequency and duration of visits by representatives from GOREL to the MKRCA buffer zone communities. This initiative aims to foster trust and narrative congruence between GOREL and the local communities. Informal knowledge exchange opportunities can arise by spending more time with community members, facilitating mutual learning, and sharing perspectives in a casual setting.
2. *Broadened Participation in Management Committee Meetings:* Expand and enhance opportunities for community participation in MKRCA management committee meetings. This step addresses the initial phase of the resilience-based framework by establishing discussion forums where community members can actively contribute to agenda-setting and decision-making processes. By creating avenues for meaningful participation, community members are more likely to engage in these meetings and perceive them as valuable.
3. *Collaborative Monitoring Initiatives:* Facilitate collaboration between GOREL representatives and local guard volunteers during patrolling trips within the MKRCA. This initiative seeks to enhance mutual understanding and appreciation of on-the-ground conservation efforts. By involving GOREL in monitoring activities, insights into the challenges and realities faced by community members can be gained, promoting shared responsibility and informed decision-making.
4. *Establishment of Comprehensive Evaluation Mechanisms:* Modify the existing semi-annual management committee meetings to allow broader participation and active engagement from all stakeholders, ensuring that progress towards the set objectives and goals within the MKRCA is assessed collectively. This step aligns with the joint process evaluation aspect of the resilience-based framework, aiming to facilitate continuous participatory evaluation and knowledge co-production. The outcomes of these evaluations can be documented and disseminated within the communities to enhance transparency and understanding.
5. *Empowerment and Recognition of Community Contributions:* Address power imbalances within the co-management system by increasing community involvement in the rule-making process and integrating Maijuna and Kichwa Traditional Ecological Knowledge (TEK) and traditional management strategies. Recognizing and utilizing this TEK not only values their everyday conservation efforts but also enhances the conservation and management of the MKRCA. This recommendation aims to mitigate power differentials, enhance community ownership, and ensure active participation in decision-making processes. By empowering community members and acknowledging their cultural and ecological contributions, we can foster a more equitable and effective co-management system that truly supports biocultural conservation.
6. *Leverage Connections with Other Government Agencies:* The MKRCA management team has access to various government branches, including the Health Ministry and the Education Ministry. By establishing inter-ministerial programs that address the comprehensive needs of the Maijuna communities, such as combining health and education initiatives with environmental conservation programs, holistic development plans can be created that benefit the communities on multiple fronts. This integrated approach will address immediate needs and build long-term trust and collaboration, fostering a more effective and sustainable co-management framework.

By adopting these approaches, the MKRCA can better align its conservation efforts with the needs and wisdom of the local communities, ensuring a more inclusive and effective management strategy for this area of enormous ecological and cultural significance.

The legal structure of RCAs, allowing direct use and economic development by local communities who also have a stake in the management decisions being made, offers a promising foundation for effective community-based conservation systems that prioritize people and biodiversity. However, the success of the RCAs in achieving their goals largely relies on the management of the area being truly collaborative, with equal engagement from local communities and regional governments. While we review the MKRCA here, we emphasize that these points of collaboration that we highlight apply across direct-use areas in the Amazon, many of which are critical for regional food security and economic stability (e.g. Coomes 2004; Mahabale et al. 2025).

The gaps that we discuss in this study also emphasize the need for continual monitoring and evaluation to be done to ensure that governance of RCAs is ethical, with equal engagement by all parties. Gaps in knowledge among community members who are active users, and target beneficiaries, of the RCA system demonstrate that there has been unequal engagement in the last decade, with significant room for improvement. Though regional government offices may have limited resources to conduct robust evaluation programs, including interviews with community members, developing greater trust and rapport with community members will contribute to increased transparency and clearer communication about barriers. In this, the recommendations we provide will act not only to improve governance of the MKRCA but provide a clear roadmap for regional government officials to build the needed rapport and open the needed lines of communication for effective governance of other areas.

## Supplementary information

Supplementary information

## Data Availability

The data generated and analyzed during this study are not publicly available in order to protect the privacy and confidentiality of study participants, in accordance with IRB requirements and the principles of Community-Based Participatory Research. De-identified interview transcripts and related materials may be made available from the corresponding author upon reasonable request and with the approval of the participating communities.
