# Supplementary material for: Indigenous Perspectives on the Co-management of a Regional Conservation Area in the Peruvian Amazon
Source: Environ Manage. 2026 Feb 20;76(4):109. doi: 10.1007/s00267-025-02310-2 (PMC12923451; doi:10.1007/s00267-025-02310-2)
Supplement: Supplementary file 1 — Supplementary information [file 267_2025_2310_MOESM1_ESM.docx]

Indigenous perspectives on the establishment and co-management of a large regional conservation area in the Peruvian Amazon

Supplementary Information

Methods

**Study Site**

The Kichwa of the Peruvian Amazon trace their ancestry back to the Quijo people of the upper Napo River basin in Ecuador (Ministerio de Cultura, 2021). Despite enduring periods of oppression, including slavery during Spanish colonization and exploitation during the rubber boom era, the Kichwa people have maintained a strong connection to their cultural heritage and language (Ministerio de Cultura, 2021). They rely on traditional practices such as hunting, fishing, farming, and foraging for subsistence and are renowned for their craftsmanship (Ministerio de Cultura, 2021). More than 23 Kichwa communities residing in the buffer zone of the MKRCA (Figure 1) participate in the MKRCA's management committee.

**Data Collection**

When assessing the perceived effects of the MKRCA establishment and subsequent co-management on Maijuna communities, the lack of pre-intervention data makes it difficult to establish a baseline for comparison. However, CBPR addresses this issue by directly engaging those who have witnessed and lived through these effects. By actively involving those directly affected by the intervention, CBPR provides a solid foundation for effective strategies to address any issues, leading to actions guided by an accurate and comprehensive understanding of the community and its needs (Huffman, 2017). Continuous collaboration and feedback from community members help in refining research questions, ensuring they align with the community's perspectives and priorities. This cultural responsiveness enhances the validity of the findings by ensuring that the research is conducted in a manner that respects and reflects the community's cultural context. As part of the research team's commitment to participating in the decolonization of scientific research, the CBPR methodology and data analysis methods used within this study were carried out under the recommendations of LaVeaux and Christopher (2009) for conducting CBPR in collaboration with Indigenous communities and the culturally responsive Indigenous evaluation (CRIE) framework principles (Bowman et al., 2015).

**Interviewing Approach**

The interview protocol followed an appreciative action inquiry (AAI) approach, as recommended to be used in CBPM by Ludema et al. (2001) since it "emphasizes the strengths of the researched" as a way to create social change (Chilisa, 2020, p. 321), primarily focusing on the Discovery, Dreaming and Design phases of AAI. Under AAI, the researcher guides the conversation with the interviewee through open-ended questions that address four different phases (Chilisa, 2020):

- Discovery, in which participants convey their history and what they most value and want to enhance about their communities.
- Dreaming, in which participants convey the type of community they envision and suggest plans for the future.
- Design, in which participants introduce possible strategies to implement these plans.
- Destiny, in which, with these strategies in mind, participants realign their activities to the vision of the community they created.

The use of these phase-specific questions was intended to allow for a deeper understanding of where the Maijuna see their community in the future, whether or not they see their values represented within the MKRCA's co-management, and the kind of culturally significant changes they think would benefit the MKRCA's co-management plans.

References

Bowman, N., Francis, C.D., Tyndall, M., 2015. Culturally responsive Indigenous evaluation: A practical approach for evaluating Indigenous projects in tribal reservation contexts. Continuing the Journey to Reposition Culture and Cultural Context in Evaluation Theory and Practice 335–360.

Chilisa, B., 2020. Indigenous research methodologies. SAGE Publications.

Huffman, T., 2017. Participatory/Action Research/CBPR, in: The International Encyclopedia of Communication Research Methods. John Wiley & Sons, Ltd, pp. 1–10. https://doi.org/10.1002/9781118901731.iecrm0180

LaVeaux, D., Christopher, S., 2009. Contextualizing CBPR: Key Principles of CBPR meet the Indigenous research context. Pimatisiwin 7, 1.

Ludema, J.D., Cooperrider, D.L., Barrett, F.J., 2001. Appreciative inquiry: the power of the unconditional positive question, in: Handbook of Action Research. pp. 189–199.

Ministerio de Cultura, 2021. Pueblo Kichwa.
